# Effectiveness of thermal screening in detection of COVID-19 among truck drivers at Mutukula Land Point of Entry, Uganda

**DOI:** 10.1371/journal.pone.0251150

**Published:** 2021-05-13

**Authors:** Andrew Nsawotebba, Ivan Ibanda, Isaac Ssewanyana, Patrick Ogwok, Francis Ocen, Christopher Okiira, Atek Kagirita, Dennis Mujuni, Didas Tugumisirize, Joel Kabugo, Abdunoor Nyombi, Robert Kaos Majwala, Bernard Ssentalo Bagaya, Simeon Kalyesubula-Kibuuka, Willy Ssengooba, Susan Nabadda

**Affiliations:** 1 Department of National Health Laboratory and Diagnostic Services, Ministry of Health, Kampala, Uganda; 2 Africa Centres for Disease Control and Prevention/African Society for Laboratory Medicine, Addis Ababa, Ethiopia; 3 Uganda National Health Laboratory Services, Kampala, Uganda; 4 Ministry of Health, Kampala, Uganda; 5 College of Veterinary Medicine, Animal Resources and Biosecurity, Makerere University, Kampala, Uganda; 6 Marascientific, Kampala, Uganda; 7 Tuberculosis and Leprosy Control Division, Ministry of Health, Kampala, Uganda; 8 Uganda National Tuberculosis Reference Laboratory /Supranational Reference Laboratory, Ministry of Health, Kampala, Uganda; 9 Department of Medical Microbiology, School of Biomedical Sciences, Makerere University College of Health Sciences, Kampala, Uganda; 10 Makerere University Lung Institute, Makerere University College of Health Sciences, Kampala, Uganda; National Institute for Infectious Diseases Lazzaro Spallanzani-IRCCS, ITALY

## Abstract

**Introduction:**

Despite the limited evidence for its effectiveness, thermal screening at points of entry has increasingly become a standard protocol in numerous parts of the globe in response to the COVID-19 pandemic. We sought to determine the effectiveness of thermal screening as a key step in diagnosing COVID-19 in a resource-limited setting.

**Materials and methods:**

This was a retrospective cross-sectional study based on a review of body temperature and Xpert Xpress SARS CoV-2 test results records for truck drivers entering Uganda through Mutukula between 15^th^ May and 30^th^ July 2020. All records missing information for body temperature, age, gender, and Xpert Xpress SARS CoV-2 status were excluded from the data set. A data set of 7,181 entries was used to compare thermal screening and Xpert Xpress SARS CoV-2 assay test results using the diagnostic statistical test in STATAv15 software. The prevalence of COVID-19 amongst the truck drivers based on Xpert Xpress SARS CoV-2 assay results was determined. The sensitivity, specificity, positive predictive value, negative predictive value, positive and negative Likelihood ratios were obtained using Xpert Xpress SARS CoV-2 assay as the gold standard.

**Results:**

Based on our gold standard test, the proportion of persons that tested positive for COVID-19 was 6.7% (95% CI: 6.1–7.3). Of the 7,181 persons that were thermally screened, 6,844 (95.3%) were male. The sample median age was 38 years (interquartile range, IQR: 31–45 years). The median body temperature was 36.5°C (IQR: 36.3–36.7) and only n (1.2%) had a body temperature above 37.5°C. The sensitivity and specificity of thermal screening were 9.9% (95% CI: 7.4–13.0) and 99.5% (95% CI: 99.3–99.6) respectively. The positive and negative predictive values were 57.8 (95% CI: 46.5–68.6) and 93.9 (95% CI: 93.3–94.4) respectively. The positive and negative Likelihood Ratios (LRs) were 19 (95% CI: 12.4–29.1) and 0.9 (95% CI: 0.88–0.93) respectively.

**Conclusion:**

In this study population, the use of Thermal screening alone is ineffective in the detection of potential COVID-19 cases at point of entry. We recommend a combination of screening tests or additional testing using highly sensitive molecular diagnostics such as Polymerase Chain Reaction.

## Introduction

Towards the end of 2019, the emergence of several cases of mysterious pneumonia was reported in Wuhan, Hubei Province, China [1–3]. Soon after, a novel type of coronavirus SARS-CoV-2 (previously 2019‐nCoV) was isolated by Chinese authorities on 7 January 2020 [1, 3]. The World Health Organization (WHO) declared Coronavirus disease 2019 (COVID-19) as pandemic on March 11, 2020 [4]. By August 15th, 2020, COVID-19 had continued to spread worldwide, causing over 21 million cases and over 755 thousand deaths. By the same time, Uganda had so far confirmed over 1,300 cases of COVID-19 with 12 related deaths since March 21^st^, 2020 when it reported its first case [5, 6].

The COVID-19 diagnosis is based on a combination of clinical symptoms and confirmed by SARS-CoV-2 PCR. Clinical features of COVID-19 are diverse, ranging from an asymptomatic state to acute respiratory distress syndrome and multi-organ dysfunction which are indistinguishable from other respiratory infections [7]. In the symptomatic patients, fever, cough, myalgia, fatigue, and shortness of breath are the predominant clinical manifestations of COVID‐19, with the disease taking on a severe course in 25% of individuals [8]. Multiple studies have reported fever as the most common symptom among patients with COVID-19 [9–14]. Studies have shown that non-contact thermal screening devices are effective at measuring body temperature [15–19].

Thermal screening at Air or Land Points of Entry was formerly implemented during the 2003 SARS epidemic and 2009 influenza A (H1N1) pandemic to limit the probability of infected cases entering other countries or regions [20–22]. In response to the COVID-19 global pandemic, thermal screening has increasingly become a standard protocol in many parts of the globe [22]. Here, we used the available evidence of the FDA approved Real-Time PCR Xpert Xpress SARS CoV-2 test results for truck drivers that were both thermal screened and tested for COVID-19 at the busy Mutukula Land Point of Entry to evaluate the effectiveness of thermal screening in detecting COVID-19 cases.

## Methods and materials

### Study design and setting

This was a retrospective cross-sectional study based on a review of body temperature and Xpert Xpress SARS CoV-2 test results records for truck drivers that were thermally screened and tested for COVID-19 at the Mutukula border point, Kyotera District in Uganda between the 15^th^ May and 30^th^ July 2020. For each driver, both thermal screening and Xpert Xpress SARS CoV-2 testing were done on the same day.

**Fig 1:**
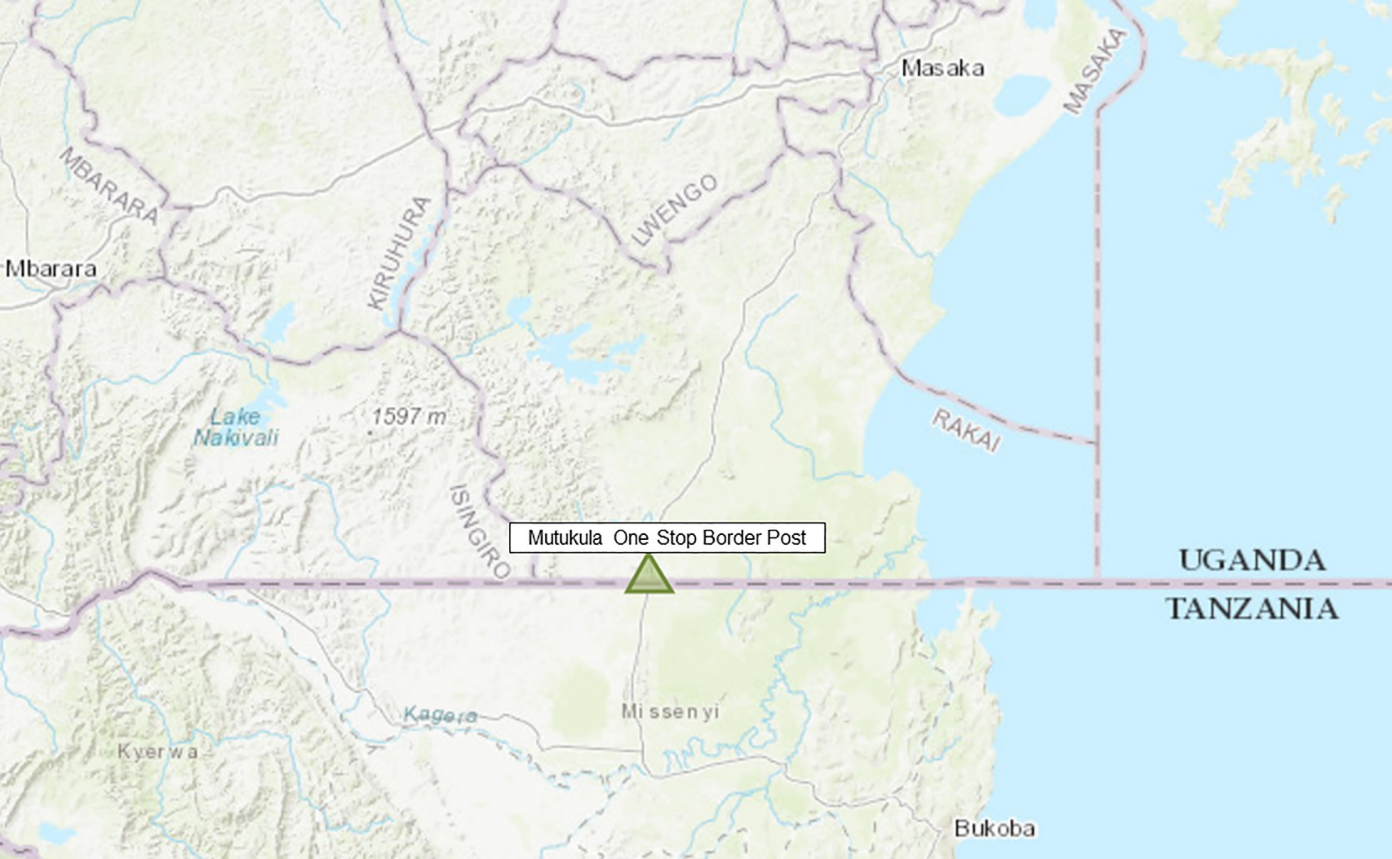
Location of Mutukula border entry point into Uganda.

Mutukula is currently one of the busiest Land Points of Entry into Uganda [23]. It is located in the extreme southern Kyotera District at the international border between Uganda and Tanzania (Fig 1). This One-Stop Border Post (1°00’00.0"S, 31°25’00.0"E) serves as the main crossing point, for both human and commercial traffic between Uganda and Tanzania.

#### Thermal screening approach

The sociodemographic information including age, gender, residence, etc. of all persons entering Uganda was captured as written in their travel documents onto the Laboratory Investigation Form (LIF). Using calibrated forehead non-contact thermometers (Non-contact Thermometer; IT– 122; Accuracy ±0.2°C; CE RoHS, China), a single body temperature for each of the persons entering the country from Tanzania was taken and recorded on top of the LIF. The truck drivers were then screened for more COVID-19 related symptoms including cough, headache, runny nose, and general body weakness. A record of the presence of any of these was taken on the LIFs.

#### Sample collection and point of care Xpert Xpress SARS CoV-2 testing approach

The sample collection was routinely done in approximately 15-minutes after the thermal and symptomatic screening. Nasopharyngeal swabs were collected using flocked swabs and were immersed in 2 mL of viral transport medium [24]. The specimens were then tested using the Xpert Xpress SARS-CoV-2 assay (Cepheid, Sunnyvale, CA) according to the manufacturer’s instruction.

### Eligibility criteria

All records corresponding to persons who had been screened for body temperature, other COVID-19 related symptoms, and also tested for COVID-19 using the Xpert Xpress SARS CoV-2 assay between 15^th^ May and 30^th^ July 2020 were included in the review for the study. Any record missing information for body temperature, age, gender, and Xpert Xpress SARS CoV-2 status were excluded from the data set.

### Ethical statement

The data used in the study was originally collected for routine care as one of the efforts to prevent and control COVID-19 transmission and therefore, the researchers had no direct patient interaction. Permission to conduct the study was sought from the Mutukula Port Health Laboratory and Uganda Central Public Health Laboratories Management. The unique identifiers for the records from May to July 2020 corresponding to truck drivers screened and tested at Mutukula PoE were cloaked before releasing the data set to the study team.

### Statistical analysis

The data received was organized and checked for completeness in Microsoft Excel and then exported to STATAv15 software for statistical analysis. This data contained information of archived Laboratory Investigation Forms for COVID-19 and results reports from which the records for body temperature, age, gender, and Xpert Xpress SARS CoV-2 status were obtained. The prevalence of COVID-19 among the persons screened and tested at Mutukula Land Point of Entry was determined as proportion positive for COVID-19 based on Xpert Xpress SARS CoV-2 assay results. The sensitivity, specificity, positive predictive values, negative predictive values, positive and negative Likelihood ratios were obtained using the diagnostic statistical test. A diagnostic test calculator [25] employed to draw the diagnostic tree and Fagan nomogram. The results of the analysis were presented in tabular and graphical forms.

## Results

### Thermal screening approach and the descriptive statistics

**Fig 2:**
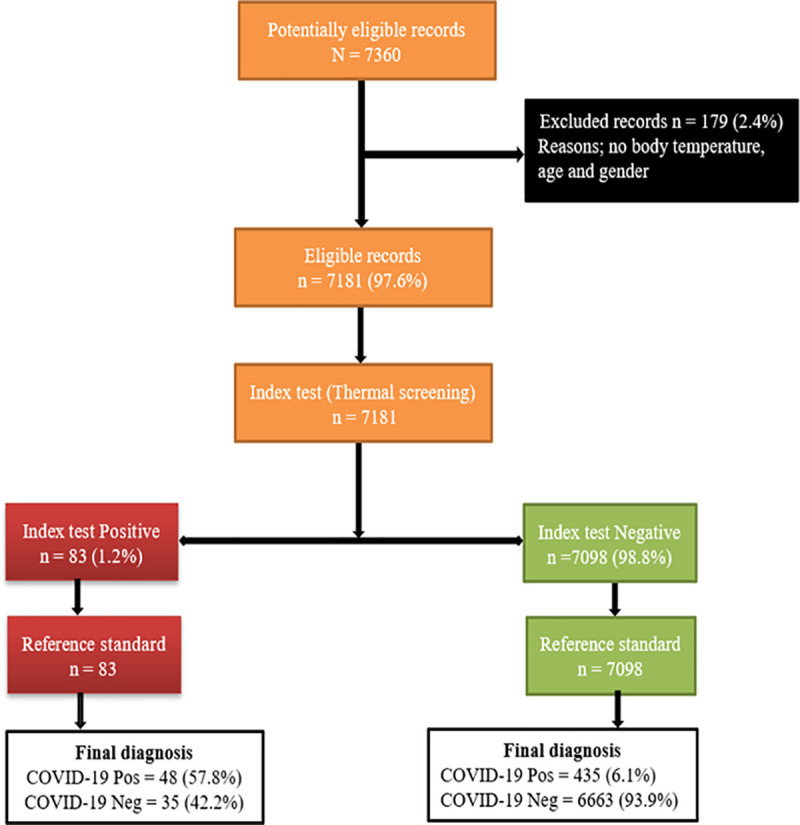
The flowchart for thermal screening in comparison to Xpert Xpress SARS CoV-2 testing.

A total of 7,630 records was received from which 179 entries were excluded due to incomplete information for body temperature, age, and gender as shown in Fig 2.

Out of the 7,181 records obtained, the median age of the truck drivers represented was 38 years (IQR: 31–45 years). The median body temperature as measured by the non-contact thermometers was 36.5 (IQR: 36.3–36.7) with only 1.2% above 37.5°C as depicted in Table 1.

**Table 1:** The descriptive characteristics of the truck drivers.

Truck drivers’ characteristic	Frequency, n (%)	
	Entire sample (N = 7181)	Temp > 37.5°C (n = 83)
Age (years)		
Median (IQR)	38 (31–45)	
Age groups (years)		
≤ 20	211 (2.9)	1 (1.2)
21 and 40	4036 (56.2)	52 (62.6)
41 and 60	2757 (38.4)	28 (33.7)
Above 61	177 (2.5)	2 (2.4)
Gender		
Male	6844 (95.3)	80 (96.4)
Female	337 (4.7)	3 (3.6)
Temperature (°C)		
Median (IQR)	36.5 (36.3–36.7)	

IQR: Inter-Quartile Range; %: Percentage; n: Number;°C: Degrees Celsius.

### Comparison of thermal screening against Xpert Xpress SARS CoV-2 assay

**Fig 3:**
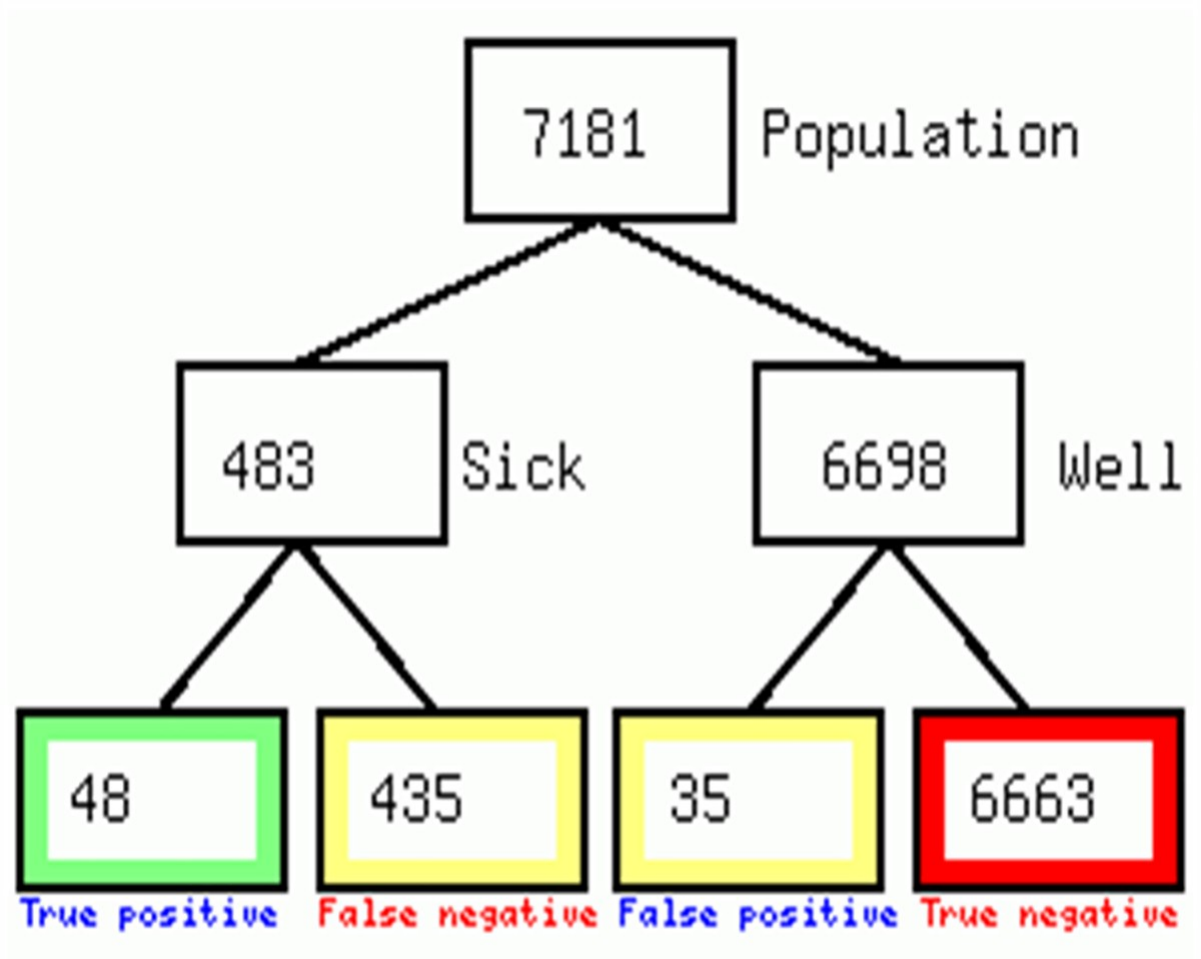
A diagnostic tree comparing thermal screening to Xpert Xpress SARS CoV-2 assay.

The proportion of persons that tested positive for COVID-19 was found to be 6.7%. The diagnostic tree (Fig 3) and the contingency table (Table 2) illustrate the performance of thermal screening at detecting COVID-19 positive truck drivers against the standard Xpert Xpress SARS CoV-2 assay. Only 48 (9.9%) out of the persons who had a positive symptom screening using forehead thermometers were found positive on Xpert Xpress SARS CoV-2 and 35 persons who had positive fever screening using forehead thermometers were negative on Xpert Xpress SARS CoV-2 from a pool of 6698 Xpert Xpress SARS CoV-2 negatives (see Fig 3).

**Table 2:** Contingency table showing results of diagnostic accuracy of thermal screening.

Thermal screening	Xpert® Xpress SARS CoV-2 Testing		Total (n)
	Positive (n)	Negative (n)_	
Positive (>37.5°C)	48	35	83
Negative (≤ 37.5°C)	435	6,663	7,098
Total (n)	483	6,698	7,181
Test characteristic	**Percentage**		**95% Confidence Interval**
Sensitivity	9.9		7.4–13.0
Specificity	99.5		99.3–99.6
Positive Predictive value	57.8		46.5–68.6
Negative predictive value	93.9		93.3–94.4

The diagnostic evaluation of thermal screening accuracy against Xpert Xpress SARS CoV-2 as the gold standard found its sensitivity, positive predictive value, specificity, and negative predictive value to be 9.9%, 57.8%, 99.5%, and 93.9% respectively, Table 2.

### Evaluation of the usefulness of thermal screening for COVID-19 at detecting cases using Likelihood ratios

The positive likelihood ratio (LR) was found to be 19 (95% CI: 12.4–29.1) and the negative LR was 0.9 (0.88–0.93) as in Table 3.

**Table 3:** The likelihood ratios (LRs) of thermal screening as a diagnostic test at POC.

Test result	Likelihood Ratio (95% Confidence Interval)
Positive	19 (12.4–29.1)
Negative	0.9 (0.88–0.93)

**Fig 4:**
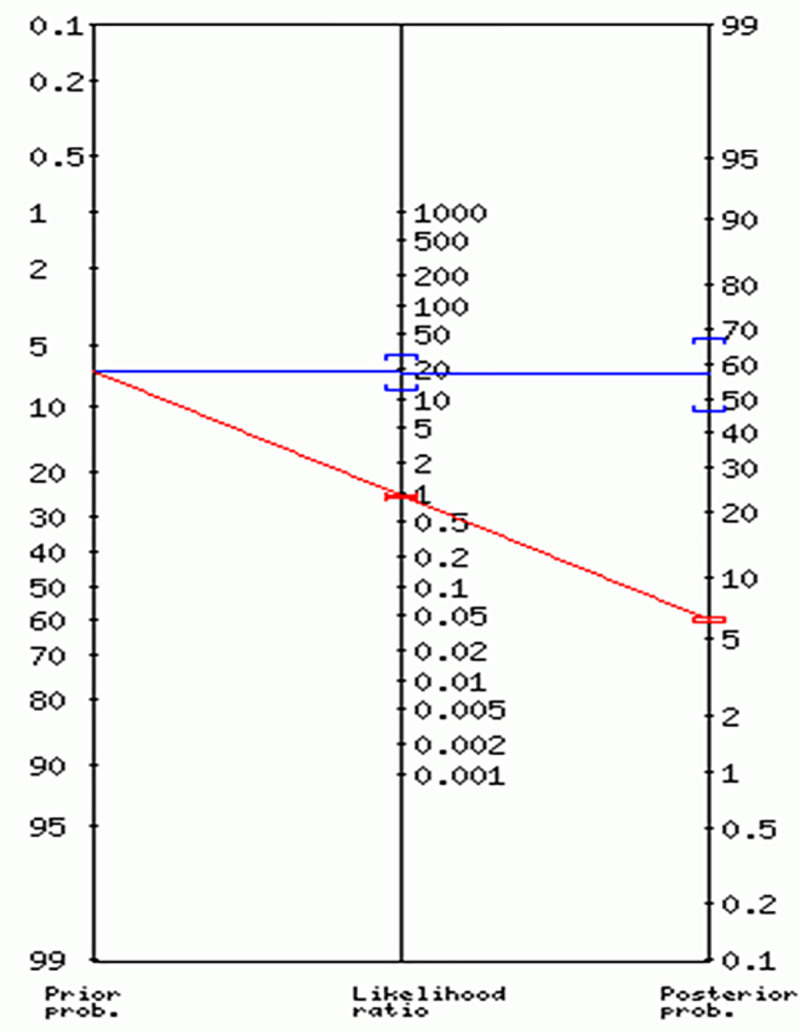
The nomogram for the likelihood ratios of thermal screening as compared to the molecular assay.

From the nomogram below (Fig 4), the prior probability of testing positive was 7%; posterior positivity probability was increased to 58% whereas the negative post-probability was decreased to 6%.

## Discussion

Our study demonstrated a significantly lower sensitivity of 9.9% (95% CI: 7.4–13.0) of thermal screening using the Xpert Xpress SARS CoV-2 test as a reference standard. This shows that only 1 in every 10 COVID-19 infected truck drivers could be detected by thermal screening. In infectious disease outbreaks like COVID 19, a good screening test should be able to pick as many positive cases as possible compared to the standard confirmatory tests. Given that fever is one of the cardinal symptoms of COVID 19, our findings are in agreement with other studies that have reported a significant number of asymptomatic cases among SARS CoV-2 infected individuals [1, 26–28]. The low sensitivity of thermal screening is a public health menace since a report in Germany indicated the ability of an asymptomatic person to transmit the virus to another [28]. However, the contribution of such asymptomatic cases in the spread of the SARS-CoV-2 is not well established and demands further investigations to ascertain the extent and role in transmission hence inform policymakers to give scientifically comprehensive recommendations. Our study revealed a high specificity of 99.5% (95% CI: 99.3–99.6) using the Xpert Xpress SARS CoV-2 test as a reference standard. This means that all those who were negative for the disease were correctly identified.

The positive predictive value (PPV) and negative predictive value (NPV) were 57.8% (95% CI: 46.5–68.6) and 93.9% (95% CI: 93.3–94.4) respectively. The PPV shows that 42 in every 100 truck drivers were detected as patients yet they were actually not diseased. The NPV indicates that 93 in every 100 non-infected truck drivers that had normal body temperature upon thermal screening were in actual sense negative upon Xpert Xpress SARS CoV-2 testing. The relatively low PPV signifies inaccuracy of thermal screening at COVID-19 detection; however, this could be higher in a sample with a higher prevalence than the 6.7%.

The high positive LR of 19 signifies that it is about 19 times more likely to observe a fever (above 37.5°C) in a COVID-19 diseased truck driver than in a healthy individual. This means that the probability of detecting a COVID-19 case using thermal screening is increased from 7% to 58%. It also implies that a positive thermal screen test is a significant indicator of a positive Xpert Xpress SARS CoV-2 test. However, the very high negative LR indicates a non-significant decrease in the probability of not detecting fever (positive thermal screening test) in a positive Xpert Xpress SARS CoV-2 truck driver in comparison to a healthy individual. That is to say, a screening test that shows normal body temperature will not significantly decrease the probability of diagnosing COVID-19 in a truck driver using Xpert Xpress SARS CoV-2 assay. This further confirms the low diagnostic accuracy of detecting positive COVID19 cases using thermal screening.

Our study provides a comprehensive description of the effectiveness of thermal screening in the detection of COVID-19 based on prior research that had identified fever as a major clinical characteristic of confirmed cases of COVID-19. Our findings are in agreement with Quilty *et al* [29] and the WHO findings that highlighted that temperature screening alone, at exit or entry, is not an effective way to stop international spread since infected individuals may be in the incubation period, may not express apparent symptoms early on in the course of the disease, or may dissimulate fever through the use of antipyretics [29].

Non-contact thermometers are operator dependent given that there are variations in the distance assumed between the gun and the forehead of the truck driver. Therefore, even though, devices have the benefits of being easy to use, easy to clean, and disinfect among others, they must be used correctly to get accurate readings.

Considering that most of the truck drivers included in the review were between 21 and 40 years and majorly males, the nature of age and gender distribution may lead to a misleading interpretation of results for which an age and gender-standardized asymptomatic proportion would be more appropriate. The presence of fever among non-infected persons may correlate with other health conditions such as malaria, tuberculosis, etc. Besides, certain disease conditions such as tuberculosis and malaria are often associated with high fevers, thus, not every fever is due to COVID-19, therefore, a more detailed data documenting the baseline health of individuals including the presence of underlying diseases would be useful to remove the bias in estimates of asymptomatic patients.

The strengths of our study are harbored on; calibration of the thermal guns, onsite sample collection, processing and testing, the gold standard test used and the type and size of population data considered. The thermal guns used to take the body temperatures were duly calibrated by the Uganda National Health Laboratory Services prior to their use at the point of entry. Training on use and care for these devices and ensuring that users adhere to the manufacturer’s instructions were always advocated for. The samples collected from the truck drivers were collected, processed, and tested immediately after the screening test to provide correlatability between the screening and gold standard test results facilitated by high sample quality and integrity and short turnaround time. The population as represented by the data used in this study was, then, at a high risk of infection with COVID-19 which provided a better estimate of the disease prevalence. The comparison of the thermal screening test was done against the routine WHO recommended and FDA approved confirmatory Xpert Xpress SARS CoV-2 test in a large sample size which increases credibility and generalizability of our findings.

## Conclusion and recommendations

Our study findings reveal that the use of the thermal screening approach alone is ineffective in the detection of COVID-19 in a resource-limited setting. We recommend further testing using highly sensitive molecular diagnostics such as Xpert Xpress SARS CoV-2 assay in the detection of the novel COVID-19 virus. Thermal screening lacks sensitivity to reliably detect COVID-19 at border points.
